# Treating anxiety in autistic adults: study protocol for the Personalised Anxiety Treatment–Autism (PAT-A©) pilot randomised controlled feasibility trial

**DOI:** 10.1186/s13063-020-4161-2

**Published:** 2020-03-14

**Authors:** Jeremy R. Parr, Samuel Brice, Patrick Welsh, Barry Ingham, Ann Le Couteur, Gemma Evans, Alexander Monaco, Mark Freeston, Jacqui Rodgers

**Affiliations:** 1 Population Health Sciences Institute, Newcastle University, Newcastle upon Tyne, UK; 2 Cumbria, Northumberland, Tyne and Wear NHS Foundation Trust, Newcastle upon Tyne, UK; 3 School of Psychology, Newcastle University, Newcastle upon Tyne, UK

**Keywords:** Anxiety, Autism, Adults, Psychological therapy, Cognitive behaviour therapy, Randomised trial

## Abstract

**Background:**

Anxiety is common in autistic adults and significantly limits everyday opportunities and quality of life. Evidence-based psychological therapies offered by mental health services often fail to meet the needs of autistic adults. The development of appropriate treatments for mental health conditions and, in particular, anxiety has been identified as a key priority by the autism community. The Personalised Anxiety Treatment–Autism (PAT-A©) trial aims to address this need by investigating the feasibility and acceptability of delivering an individualised psychological treatment for anxiety experienced by autistic adults.

**Methods/design:**

This is a pilot randomised controlled feasibility trial. Up to 40 autistic adults with clinically diagnosed anxiety will be randomised into one of two groups (either the PAT-A© intervention or Current Clinical Services Plus two emotional literacy skills sessions). Before randomisation, participants will receive a detailed clinical assessment to inform formulation and guide anxiety treatment. As part of the baseline assessment participants will also identify two personally important ‘target situations’ that cause significant anxiety and impact upon their daily life. Based upon the formulation and identified target situations, participants randomised to the PAT-A© intervention will receive up to 12 individualised, one-to-one therapy sessions. Initial emotional literacy training sessions will be followed by a bespoke, modular, needs-based treatment approach utilising one or more of the following approaches: Mindfulness, Coping with Uncertainty in Everyday Situations (CUES), social anxiety and graded exposure within Virtual Reality Environments. Participants in the control arm will receive two psycho-educational sessions focussing on understanding and describing emotions and be signposted to healthcare provision as required. Data will be collected through quantitative and qualitative methods.

**Discussion:**

This feasibility pilot trial serves as the first stage in the development and evaluation of a manualised personalised, evidence-based psychological therapy treatment for anxiety in autistic adults. Study outcomes will be used to inform an application for a fully powered multi-site intervention trial of adults and young people.

**Trial registration:**

ISRCTN, ID: 15881562. Retrospectively registered on 9 August 2019.

## Background

Autistic people experience difficulties with social communication, social interaction and restricted, repetitive patterns of behaviours [1]. Mental health conditions are significantly more prevalent in autistic adults compared to the general population [2, 3]. In particular, estimates of clinically significant anxiety over the lifespan indicate a prevalence of between 30 and 60% in samples of autistic adults [4–6]. Anxiety presentations in autistic people, however, can vary and may have a distinct presentation, which may be underpinned by the individual’s autism-related profile. The symptoms may include social discomfort not associated with a fear of negative evaluation; engagement in compulsive behaviours that are not motivated by distress relief or idiosyncratic fears and phobias; and significant difficulties coping with change or uncertainty [7].

Recent research studies using the World Health Organisation International Classification of Functioning (IC F; [8] highlight that the impact of autism and co-existing mental health conditions extends far beyond core symptom domains to include personal interests/activities, engagement with peers, family members and the local community, as well as other areas of physical and mental wellbeing [9, 10]. The range and frequency of life disadvantages for autistic adults (across the socio-economic spectrum, including education, employment, finances, social services, criminal justice system contact, and victimisation across the life-course) has been reported in a recent study [11]. Autistic adults have reported significantly more disadvantages than non-autistic adults and these disadvantages significantly mediated the association between an autism diagnosis and depression, anxiety, and life satisfaction. Furthermore, data from the Adult Autism Spectrum Condition Cohort–UK (ASC-UK; Brice et al., what do autistic people tell us about their anxiety and treatments and services recieved? Findings from a large UK survey, in preparation) suggests that anxiety significantly limits education and employment opportunities and reduces quality of life.

In spite of increased interest in understanding the mental health needs of autistic adults [12], data from various sources indicates that health services are not currently meeting autistic adults’ needs [13, 14]. In the UK, National Health Service (NHS) mental health teams currently provide treatment for a range of anxiety disorders with cognitive behavioural therapy (CBT) offered as the predominant intervention [15, 16]. Evidence-based CBT approaches include a core set of features such as Socratic questioning, guided discovery and collaborative empiricism. These features rely on interaction with a therapist, flexibility in thinking and emotional literacy, all of which autistic people may struggle with [17]. Furthermore, some CBT techniques may be difficult for people with autism, such as the ability to change behavioural routines and identify how to modify cognitive processes [18]. Difficulties with imagination [19] mean that standard techniques, such as imaginal exposure, may not always be accessible, without modification. All of these features can impact upon engagement and establishing a therapeutic relationship, hence the need for bespoke personalised approaches to the delivery of psychological therapies specifically developed to address the anxiety experienced by autistic people that are in addition to reasonable adjustments to support access to these therapies [20, 21].

Recognition of anxiety in autistic individuals may be limited as a consequence of factors such as alexithymia (difficulties recognising, understanding and describing emotions) and/or limited social communication skills [22]. In addition, autistic individuals often experience symptoms of a range of anxiety disorders concurrently [23]. This means that existing CBT packages designed for specific anxiety diagnoses in the general population may not be appropriate if specific aspects of the individual’s anxiety profile are not addressed and/or the treatment plan does not take sufficient account of the interaction between anxiety and autism. Many current psychological treatments are not always/routinely provided within a setting sufficiently adapted for people with autism or with reasonable adjustments that would facilitate access (Brice et al., in preparation). These factors partly explain why currently available CBT interventions may not be accessible and appropriate for many autistic adults presenting to primary care and mental health services [16]. The implications are that anxiety treatments should be designed and personalised for autistic adults in order to facilitate access to treatment and maximise the chances of effectiveness.

Understanding and designing appropriate autism-informed psychological therapies for mental health conditions, and, in particular anxiety, are recognised key priorities for autistic individuals [24] and for the autism research community (first and fourth in the 2016 Top Ten research questions from the Autistica-led James Lind Alliance Priority Setting Partnership; [25]). In addition, both the national adult autism strategy [26] and National Institute for Health and Care Excellence (NICE) clinical guidelines [27] highlight the prioritisation of developing high-quality, effective psychological interventions for autistic adults.

The majority of published research on the development of psychotherapies for anxiety in autism has focussed upon children and adolescents [28]. Some studies with adults have focussed on a range of intervention approaches, methods and measures of therapeutic change in a small number of single case reports [29–31]. One single-blind randomised trial of group CBT for autistic adults, found no difference in anxiety at follow-up between those allocated to treatment or a waiting-list control [32]. Novel interventions combining Virtual Reality Environments (VREs) and CBT for the treatment of specific phobias have demonstrated feasibility and acceptability and some evidence of acceptability and efficacy in autistic adults [33]. Trials of mindfulness-based psychotherapies have also obtained favourable outcomes [34, 35], for a range of symptoms such as anxiety, depression and rumination. Other trials of CBT for autistic adults similarly aim to improve emotional regulation and functioning as opposed to working specifically with anxiety-based presentations. Given the growing evidence that difficulty in tolerating uncertainty is associated with the development and maintenance of clinically significant anxiety in autistic adults [36–39], interventions aimed at increasing the ability to cope with uncertainty have been piloted for feasibility and have demonstrated promise as a treatment option [40].

The Personalised Anxiety Treatment–Autism (PAT-A©) randomised feasibility trial is designed to investigate the feasibility and acceptability of delivering a manualised individualised anxiety treatment package for autistic adults with a variety of anxiety presentations. This formulation-driven and personalised psychological approach to therapy aims to provide treatment for the diverse and often co-existing nature of anxieties found in autistic adults whilst accommodating the specific needs and personal preferences (e.g. sensory reactivity, social motivation, approaches to learning) of the individual.

## Methods/design

### Study design

This study is a pilot feasibility randomised controlled, single-centre trial (pilot RCT) with a blind-to-group outcome assessor. The trial has two arms; an intervention arm (Personalised Anxiety Treatment–Autism; PAT-A©) and a control arm (Current Clinical Services Plus; CCSP). Adults aged 18 years or above with a clinical diagnosis of an autism spectrum disorder and at least one clinically recognised anxiety disorder that may benefit from psychological therapy will be included. All recruited participants will live in North East England and have accessed care from a local NHS mental health trust.

### Aims and objectives

This is a feasibility trial, hence recruitment, acceptability, retention, completion of outcome measures and adherence of participants to the two study arms (PAT-A© and CCSP) will be assessed as the main outcomes. Other outcomes, such as the potential reduction in anxiety and increased engagement in ‘target situations’ (determined collaboratively by the participant and therapist) will also be measured to inform future trial design.

### Study registration and ethical approval

A favourable ethical opinion was provided by NHS Health Research Authority and a Research Ethics Committee (Wales REC 5, IRAS ID: 235805). Brief eligibility procedure and written informed consent will be obtained from all individuals by the research associate (RA) prior to baseline assessment.

### Sample size

This pilot feasibility trial is not powered to estimate a target difference in relative effectiveness, but rather to address feasibility and acceptability of delivering an individualised anxiety treatment package for autistic adults with a variety of anxiety presentations to inform a future definitive trial. We aim to recruit up to 40 participants. This number is based on recommendations for good practice in feasibility studies [41].

### Recruitment and eligibility criteria

Participants will be recruited from clinical teams within Cumbria, Northumberland, Tyne and Wear NHS Foundation Trust (CNTW). Clinical teams that treat people who meet the eligibility criteria described below will be approached by the RA who will outline the trial to them and explain the recruitment procedures (adult mental health and autism diagnostic services). Clinicians will be encouraged to screen their caseload and waiting list for autistic people who meet eligibility criteria before briefly discussing the trial with them and returning an Expression of Interest Form to the RA. The RA will then contact the prospective participant with full information about the trial. If, after reading this information, they are still interested, the RA will arrange a meeting to discuss the trial in more detail, answer any questions that the participant might have and, if appropriate, take written informed consent (although participants can take up to a week to decide whether they would like to take part following this meeting). The Consent Form asks participants to confirm in writing that they understand what their participation in the study will involve, understand their rights as a participant (e.g. right to anonymity, right to withdraw, how their data will be handled) and that healthcare professionals involved in their care may be informed of their participation. As a viable fall-back strategy, participants can also be recruited via the Adult Autism Spectrum Cohort–UK (ASC-UK); a large, longitudinal research cohort of autistic adults, many of whom have previously expressed an interest in taking part in research. Monitoring of trial progress (including recruitment) will be discussed at fortnightly meetings including co-investigators, RA and, where appropriate, trial therapists.

Inclusion criteria for participation in the trial include: an autism spectrum diagnosis given by an NHS clinical team; aged 18 years or older; resident in the Northumberland, Tyne and Wear area; an ability to provide informed consent and the verbal comprehension skills required to participate in interviews, talking therapy and the completion of questionnaires. Participants will also be identified as experiencing clinically significant anxiety by an NHS clinician. Intellectual ability will not be formally assessed so the trial is in line with NHS clinical practice where clinicians take pragmatic decisions about treatment and people’s abilities to engage in treatment, rather than basing treatment access on cognitive assessment scores. Participants with co-existing physical and/or mental health conditions will not be excluded unless these conditions significantly affect capacity to engage in the psychological therapy delivered as part of this study. Participants will not be excluded on the basis of having received previous or ongoing pharmacological treatments for anxiety.

**Fig. 1:**
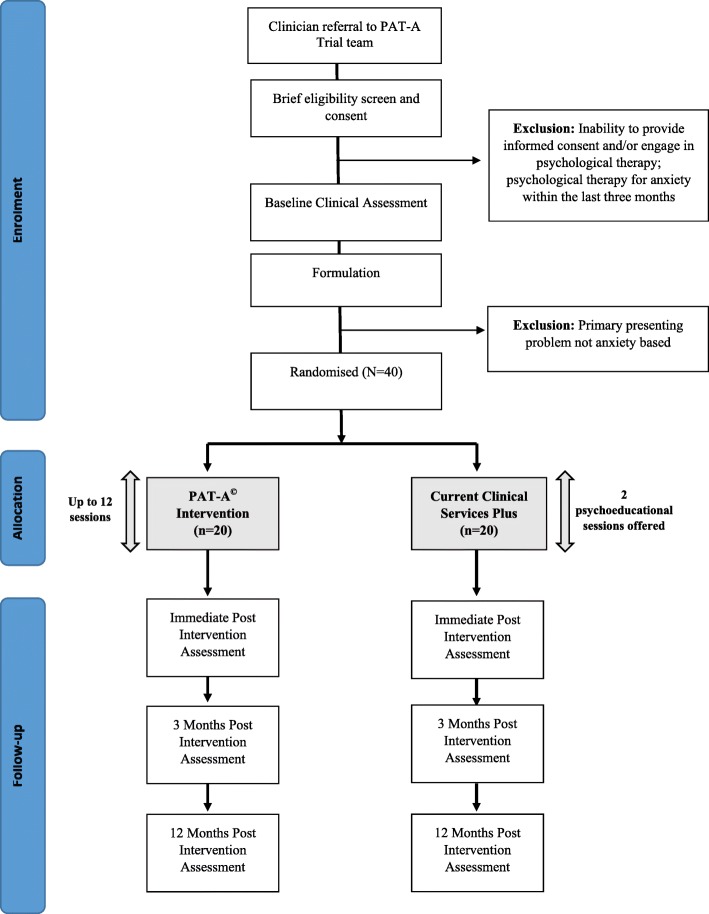
Personalised Anxiety Treatment–Autism (PAT-A©) Consolidated Standards of Reporting Trials (CONSORT) Flow Diagram

Exclusion criteria for participation in the trial include: people without an autism diagnosis; people under the age of 18 years; those already engaged in an ongoing psychotherapeutic treatment for anxiety (in the 3 months prior to consent); people who are experiencing a physical or mental health condition that would significantly impact on their ability to engage in psychological therapy for anxiety; people unable to provide informed consent (Fig. 1).

### Randomisation

All consented eligible participants who have completed baseline assessment will be randomised following the formulation and treatment planning meeting with the study team, to either the intervention (PAT-A©) arm or the control (CCSP) group via computer-generated sequence blocks (Sealed Envelope; www.sealedenvelope.com/). A simple randomisation schedule will be used without blocking or stratification as this will allow us to address the aims of the trial. A research administrator independent of the trial will oversee this process, ensuring that the RA remains blinded to the outcome of randomisation. One of the trial therapists (PW) will inform the participant of the outcome of randomisation and this will also be recorded in the participant’s NHS electronic patient record. Due to the design and configuration of this trial, it is not possible for participants to remain blinded to group; however, data analysis will be conducted by the RA who is blind to group.

### Study interventions

#### PAT-A© intervention

Taking account of the formulation and treatment plan, participants will be offered a bespoke intervention package utilising one or more manualised PAT-A© modules. All participants will receive a manualised Understanding and Describing Emotions (UaDE) module, which aims to develop a person’s ability to understand emotional states, how different situations and thoughts impact upon these states, and assessing current emotional-regulation strategies. Ensuring that individuals have the requisite knowledge and language in order to engage in conversations about physiological arousal and emotions during therapy has been highlighted as a key adaption when using CBT with autistic adults [17]. The module length depends on the participant’s pre-existing knowledge and ability in relation to emotions. The module is a scaffolding approach to enable the participant to understand other PAT-A© treatment modules.

Following on from the UaDE module, the participant will receive an intervention including one or more of the following manualised treatment modules (Fig. 3). All modules have been designed to stand alone but can also be used in combination where appropriate.

##### Mindfulness

Mindfulness-based therapies have demonstrated effectiveness for a range of mental health conditions [42] and have been adapted for use with autistic adults [34, 35, 43]. This module aims to provide individuals with skills to enable them to tolerate emotional distress in everyday situations and/or facilitate engagement with anxiety-provoking situations and behavioural experiments outlined in other treatment manuals of the PAT-A© intervention programme. The Mindfulness module has been designed to include a Mini Mindfulness approach (two to three sessions in duration) and a Full Approach (six to eight sessions) depending upon the needs of the participant.

##### Coping with Uncertainty in Everyday Situations (CUES-A©)

Intolerance of Uncertainty (IU) has been proposed as an important trans-diagnostic mechanism in the development of anxiety in autistic adults [22]. The focus of the CUES-A© intervention programme [40, 44] is to support participants to develop their awareness, gain confidence in their ability to recognise and manage their own IU through collaborative CBT strategies.

##### Social anxiety

Although autistic adults may experience fear and anxiety in social situations for a variety of reasons, this module is designed for those where social-evaluative concerns are the predominant difficulty. It contains the main elements commonly used in recommended treatments for social anxiety [45] that have been adapted for autistic adults; for example, ensuring that any social communication difficulties have been assessed and taken into account.

##### Virtual Reality Environment-delivered treatment for phobias

The Virtual Reality Environment (VRE) intervention programme uses visual images projected onto the walls and ceilings of a screened room over four 20-min sessions. Participants interact with and navigate through a specific phobia scenario with the support of a therapist. Feasibility studies support the use of this VRE as an acceptable and effective treatment for specific phobia in both autistic young people and adults [33, 46, 47].

#### CCSP (control arm)

Participants will be offered two psycho-educational sessions focussing on understanding and describing emotions. They will then be signposted to services that are available to them as part of usual care.

### Baseline characterisation

Following informed consent taken by the RA, initial assessment measures will be completed with the participant. The main initial assessment will be the Anxiety and Related Disorders Interview Schedule for *DSM-5*: Adult Version (ADIS-5; [48]) undertaken with the participant and administered by the RA (a trained clinical psychologist). This structured interview is designed to identify current anxiety, mood, obsessive-compulsive, trauma and related disorders (e.g. somatic symptoms, substance use) according to the *Diagnostic and Statistical Manual for Mental Disorders, 5th edition* (*DSM-*5) criteria [1]. In addition, the trial team has developed an additional Summary Form to capture specific phenomena that research indicates may be particularly related to anxiety experienced by autistic people (e.g. the presence of sensory sensitivities, IU). The ADIS-5 also records historical information regarding previous or concurrent anxiety-related treatments and significant life events.

Following the anxiety assessment, the RA and participant will jointly identify and agree two personally important target situations that cause significant anxiety and impact upon the participant’s everyday functioning and quality of life. For both self-identified targets the participant will be asked about the situation that is associated with anxiety including frequency, their reactions to that situation (symptoms and intensity), whether they worry about the situation in advance, and how it interferes with daily functions and activities. These details will be summarised by the RA in a written vignette. The protocol was developed by the Research Units on Paediatric Psychopharmacology (RUPP) Autism Network [49] and has demonstrated high levels of agreement between expert raters. Arnold et al. [49] reported an Intraclass Correlation Coefficient (ICC) of 0.895 across a panel of five raters. This has been a preferred outcome measure in our previous trials, and it has demonstrated utility in recording change in anxiety associated with a specific real-life goal or situation [46, 47, 50–52].

The RA will also complete the: Session Engagement Form (developed by the research team) This form requires the RA to consider the participant’s engagement in the assessment process using five key markers, each rated on a 4-point scale: (1) level of verbal communication, (2) focus/attentiveness, (3) understanding of key concepts, (4) ability to flexibly move between topics and (5) speed of processing information.

A set of self-report measures will also be completed during the baseline assessment to enable characterisation of the individual’s autism profile and their anxiety. These include:

- Social Responsiveness Scale (SRS-2; [53]: the SRS-2 is a standardised self-report questionnaire used extensively to rate the social communication difficulties of autistic adults and children. Estimates of internal consistency of between .94 to .96 have been reported whilst the predictive validity of the adult form found a specificity level of .60 and sensitivity of .86 [54]
- Anxiety Scale for Autism–Adult (ASA-A©; [55]): the ASA-A© is a 20-item self-report measure designed to measure anxiety in autistic adults. It consists of four subscales: Social Phobia (SP), Anxious Arousal (AA) and Uncertainty (U). The ASA-A© can be summated to provide a total anxiety score, as well as broken down into subscale scores (the sum of items within a subscale). All items are rated on a 4-point Likert scale, from 0 (‘never’) to 3 (‘always’). The ASA-A© was adapted from the Anxiety Scale for Children Autism Spectrum Disorder (ASC-ASD©; [56]) in collaboration with professionals and autistic adults, to make it suitable for use in an adult population. The authors indicate that a total score of ≥ 28 on the ASA-A© may indicate the presence of significant levels of anxiety. At present, indicative cut-offs are not available for the subscales. Preliminary analysis of the psychometric properties of the ASA-A© indicate excellent internal consistency (Cronbach’s α total score = .899, Uncertainty subscale = .834, Anxious Arousal Subscale = .845, Social Phobia Subscale = .847), excellent 1-month test-retest reliability (.823) and robust convergent and divergent validity [55]
- Hospital Anxiety and Depression Scale (HADS; (Zigmond AS, Snaith RP. The Hospital Anxiety and Depression Scale, submitted)): the HADS is a 14-item self-report questionnaire widely used within a variety of community and psychiatric populations in order to measure symptoms of anxiety (seven items) and depression (seven items). Research demonstrates promising internal consistency (HADS-A, *α* = 82–.84; HADS-D, .60–.72) and convergent validity (WEMWBS, *r* = −.45 to −.60, *p* < .001; SDQ emotional scale, *r* = .80, *p* < .001; *DSM-5* DAS anxiety scale, *r* = .71, *p* < .001 and the PHQ-9 depression scale, *r* = .56, *p* < .001) when used with autistic individuals [57]
- WHO Quality of Life-BREF (WHOQOL-BREF) and Disabilities module plus Autism Addendum: the WHOQOL-BREF [58] is an abbreviated 26-item questionnaire covering four domains of quality of life (Physical health, Psychological health, Social relationships, and Environment). It is currently the most commonly used quality of life measure within autistic adult populations [59]. In order to ensure relevance to our participant group the WHOQOL-BREF will be used alongside the WHO Disabilities module [60] and the Autism Addendum recently developed by our group [61]. Internal consistency for this measure is excellent (*α* = 0.93) whilst the structural validity of the WHO factor structure is acceptable for use with autistic people (CFI = 0.902, GFI = 0.886, RMSEA = 0.059, PCLOSE = 0.006, *χ*^2^ = 519.90, *df* = 242, *p* < 0.001) [61]
- EuroQoL 5 dimensions, 5 levels health survey (EQ-5D): the EQ-5D [62] is a generic measure of health-related quality of life allowing for the calculation of Quality Adjusted Life Years (QALYs), commonly used by the NHS to measure treatment effectiveness. There is evidence that the measure demonstrates convergent validity with all items of the World Health Organisation-Five Well-Being Index (WHO-5; [63])
- Toronto Alexithymia Scale-20 (TAS-20; [64]: the TAS-20 is one of the most commonly used measures of alexithymia comprising of three subscales covering difficulty describing feelings, difficulty identifying feelings, and externally-orientated thinking. It has demonstrated test-retest reliability (*r* = 0.79–0.92), convergent validity with other measures of alexithymia such as the Bermond and Vorst Alexithymia Questionnaire-form B (BVAQ-B; *r* = 0.48–0.77, *p* < 0.05) and discriminant validity, over time, in a sample of autistic adults and controls (time 1, Exp(B) = 1.22, CI (1.09–1.36), *p* < 0.001; time 2, Exp(B) = 1.17, CI (1.06–1.29), *p* < 0.01) [65]
- Waisman Activities of Daily Living Scale (W-ADL; [44]: the Waisman Activities of Daily Living Scale (W-ADL) is a measure of independence validated in people with a broad range of developmental disability diagnoses. Analysis indicates that it is reliable over time (*κ* = 0.92–0.93), with high levels of internal consistency across disability groups (Cronbach’s *α* = 0.88–0.94) and criterion validity when compared to the Vineland Screener Adaptive Behavior Composite Score (*r* = 0.78)
- Reasonable adjustments: an adaptation of an existing checklist, will be utilised. As autistic adults state that they would like professionals to take into account their sensory sensitivities or neurological differences [12, 13], we will aim to accommodate these within the intervention by asking participants as part of the baseline characterisation about their preferred means of communication, factors which may make attending sessions easier/more difficult, current emotional-regulation techniques, special interests and considerations regarding the involvement of family members, friends or independent advocates

### Formulation of anxiety and treatment planning

Once the assessment is complete, the RA will compile a standardised assessment report including the following information:

- Current (and, where relevant, historical) mental health and physical health diagnosis(es) including severity scores from the ADIS-5 diagnostic interview
- Relevant personal details/background, the impact autism and anxiety may have on the current functioning and a summary paragraph of each anxiety/mental health disorder
- SRS-2 and TAS-20 summary scores
- A summary of reasonable adjustments required for therapy
- Two target-situation vignettes
- The Session Engagement Form

The assessment report for each consented participant will be discussed at a meeting of trial therapists and senior members of the research and clinical team convened by the RA. During this meeting, a formulation of the participant’s current anxiety-related difficulties will be developed and an individualised treatment plan agreed prior to randomisation. Decisions regarding treatment planning will be informed by a treatment algorithm developed by the clinical team as part of the trial. The treatment algorithm uses the assessment information and the participant’s target situations to plan treatment using a combination of the treatment modules available. Following the answering of any questions on the presentation, the RA will leave the room, and the clinical team will agree a treatment plan. In addition, as part of the evaluation of the feasibility of the formulation and treatment planning procedures, prior to the meeting, the RA will use the treatment algorithm to identify the treatment plan that they suggest, and seal this in an envelope to be opened at the end of the formulation meeting. Levels of agreement in treatment plans between the RA and the clinical team will be calculated; however, for the purpose of this pilot RCT the clinical team’s decision will be final.

### Feasibility and acceptability outcomes

The primary outcomes for this feasibility and acceptability pilot RCT will be recruitment (number of expressions of interest, how many of those expressing interest go on to consent (and, if not, why not?), how many are excluded and the reasons for this), acceptability (including acceptability of the randomisation procedure), retention, completion of outcome measures and treatment adherence. Treatment adherence will be measured by recording the percentage of sessions that each participant attended, compared with those scheduled and reporting the median, minimum and maximum for the sample. As treatment is individualised and, therefore, variable in length, there is no fixed number of sessions for a ‘complete’ dose. The therapist will also record each participant’s engagement in homework tasks (a core component of CBT) at each session. The participant’s engagement will be rated on a 5-point scale ranging from ‘not attempted’ to ‘extra homework completed’. Each participant’s engagement in homework will be combined into an average score over the course of their treatment and the median, minimum and maximum at sample level will be reported.

An opportunity to participate in a semi-structured qualitative interview will be offered to all participants 3 months post intervention, with the aim of gaining information about the acceptability of the trial and treatment processes from the participant’s perspective. Topics to be covered will include the acceptability of randomisation, feedback on both the PAT-A© intervention and CCSP and the appropriateness of baseline and outcome measures used. Participants will also be asked if they have experienced any changes (either positive or negative) in any other anxiety-related situation not otherwise covered in the target situation(s).

### Other outcomes

As the PAT-A© intervention has been developed to improve engagement with the specific target situations, the target situation rating will also be considered as an outcome measure to identify symptom change over time. As previously described two target situation vignettes will be developed in collaboration with the participant using baseline assessment data. Both vignettes and the severity of difficulties within the target situation are then reassessed 3 months post intervention by the RA. Degree of improvement will be rated by an expert panel of researchers and clinicians, blinded to the treatment group, which will compare the anonymised vignettes from baseline and follow-up and identify whether there has been any change since baseline. A 9-point scale (from 1 ‘normalised’, 2 ‘markedly improved’, 3 ‘definitely improved’, 4 ‘equivocally improved’, 5 ‘no change’, 6 ‘equivocally worse’, 7 ‘definitely worse’, 8 ‘markedly worse’ to 9 ‘disastrously worse’) will be used to rate change between baseline and 3 months post intervention. Comparisons of change scores for the two vignettes between the two groups across time (baseline to 3- month follow-up) will then be made. The research team also plans to repeat the target-situation interview and ratings at 12 months post intervention.

In addition to the target situations, the following questionnaire data will be collected at 3 months post intervention to allow analysis of differences between baseline and follow-up across the intervention and control groups:

• Anxiety: assessed using the full-scale score on the ASA-A
• Quality of life: assessed using full-scale score and subscale scores on the WHOQoL-BREF plus the ASQoL and disabilities module
• Health-related quality of life: assessed using full-scale score on the EQ-5D

The Clinical Global Impression of Improvement (CGI-I) will be used to assess global clinical change between baseline and 3 months post intervention. The CGI-I is a standardised framework for clinicians to assess to what extent participants’ symptoms have improved or worsened using a 7-point scale (1 – very much improved; 2 – much improved; 3 – minimally improved; 4 – no change; 5 – minimally worse; 6 – much worse; or 7 – very much worse). The RA, who is blind to group and clinically trained, will rate global improvement using all available data from baseline and 3-month post-intervention follow-up, including:

- ASA-A
- WHOQoL-BREF plus ASQoL and disabilities module
- EQ-5D
- HADS-D (depression subscale)
- Target-situation vignettes
- Responses to the interview question about change in an anxiety-related situation not otherwise included in the target-situation vignette(s)

Ratings of 1 (very much improved) and 2 (much improved) are regarded as significant ‘improvement’, ratings of 7 (very much worse) and 6 (much worse) are regarded as significant ‘worsening’ and ratings of 3–5 (inclusive) are regarded as ‘no change’. A percentage of the participants’ CGI-I score will be independently rated by a second rater to assess inter-rater reliability.

**Fig. 2:**
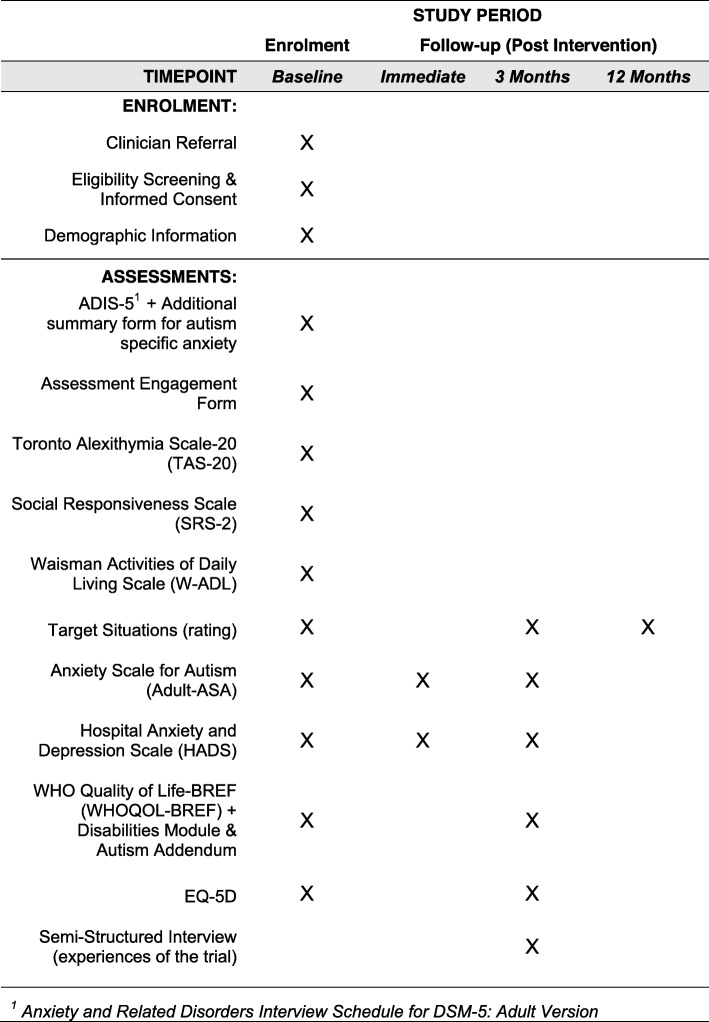
Standard Protocol Items: Recommendations for Interventional Trials (SPIRIT) Figure: schedule of enrolment, intervention and assessment within the Personalised Anxiety Treatment–Autism (PAT-A©) trial

Full details of the measures repeated at follow-up can be seen in Fig. 2.

### Data collection and management

Baseline assessment and follow-up measures will be collected by a RA blinded to the outcome of randomisation. All participants will be allocated a unique number that will be used to identify them on all paper assessment forms throughout the trial. All data collected on paper will be inputted into a statistical analysis software system and all identifying data will be stored separately. A proportion of the data recorded on paper will be inputted electronically by two researchers independently to measure data-entry error rate. Missing questionnaire data at item level will be managed according to the manual for each questionnaire. If no manual exists, any published precedents will be consulted (e.g. [66]), or the mean imputed score for that subscale/full-scale (as appropriate) will be used, providing that no more than 30% of the data is missing. All study data will be treated in accordance with the latest Directive on Good Clinical Practice (2005/28/EC).

A Data Monitoring Committee (DMC) will not be required as this is a pilot feasibility trial. The RA will discuss challenges with data collection with the lead investigators. The RA will be responsible for ongoing review of data completeness and any concerns will be discussed within the wider research team and the sponsor as appropriate.

### Treatment fidelity and engagement

The PAT-A© intervention has been designed to be delivered by clinical psychologists or suitably qualified CBT therapists with experience of working with autistic adults. Therapists will receive supervision every 2 weeks, alongside monthly group supervision and access (as and when required) to a consultation group of researchers and clinicians (comprising experts in CBT and adapting interventions for autistic people). A Session Recording Form will be completed by the therapist after each therapy session in order to track the treatment modules and specific components received by the participant and to assess therapist adherence to core CBT techniques (e.g. guided discovery, ‘homework’ setting). Where possible, therapy sessions will be audio-recorded in order to undertake independent fidelity ratings after all participants have been treated. Members of the research team will review audio-recorded sessions to ensure fidelity to the treatment module(s) as they are defined in the manual(s).

The session engagement checklist used during assessment (described above) will also be used after each therapy session to assess and record the participant’s engagement with treatment and to facilitate therapeutic review including impact of any required autism informed adjustments to therapy, or additional adjustments required.

### Modification or discontinuation of study interventions

A primary intervention approach is endorsed during the PAT-A anxiety formulation; however, this may be modified in light of new information and progress during therapy. To ensure that the intervention can be implemented, specific components of another module will be considered within the context of a developing formulation during therapy supervision between the therapist and clinical supervisor. When an entirely different intervention module is indicated (a deviation from the original PAT-A formulation), a consultation group, consisting of members of the trial team, will be convened. The recommendations of the consultation group will be recorded, although treatment decisions remain with the therapist and clinical supervisor.

Participants will be entitled to discontinue their treatment at any time and without having to give reason. Interventions may also be discontinued when both the therapist (in consultation with the clinical supervisor) and participant agree that continuing with therapy is unlikely to be beneficial due to factors such as concurrent life events, participant motivation, session attendance and engagement. Treatment will be discontinued if a screening error becomes apparent during treatment, e.g. the identification of a clinical presentation that meets the exclusion criteria or if a significant or immediate clinical risk is identified, and continuation of PAT-A would be contraindicated. Such decisions will be made through clinical supervision and in consultation with the participant. In any such eventuality, the trial therapist will ensure that the participant is referred to an appropriate service for ongoing clinical support.

### Adverse event recording

Any adverse event (defined within the trial as unexpected events) will be recorded using a pre-existing checklist [67]. Events to be recorded include deterioration of existing symptoms, the emergence of new symptoms, significant changes in life circumstances and strains in family relationships. All adverse events will be discussed in supervision and the Trial Management Group for those defined as significant will be dealt in accordance with relevant NHS protocols and reported to the sponsoring NHS Trust and the reviewing Regional Ethics Committee as per the standard operating procedure of the sponsoring NHS Trust. All adverse events will be recorded and filed in the Trial Management File (TMF). Any significant adverse events that occur as a result of the trial intervention(s) will be reported in any publications and the same may be true for an adverse event that was not caused by study interventions but likely had a significant impact on a participant’s capacity to engage successfully with trial interventions.

The trial has been indemnified by Newcastle University and Cumbria, Northumberland, Tyne and Wear NHS Foundation Trust. It is not expected that participants will experience any significant harm as a result of taking part in this trial. Should any participant experience significant harm then this would be reported to the sponsoring trust, which would manage this through their standard procedures.

### Statistical analysis

As this is a feasibility and acceptability pilot study, analyses will be mainly descriptive. Formal power calculations are not appropriate as the study is not designed to test for a difference between treatments. Feasibility will, therefore, be explored by examining:

- The number of expression-of-interest forms received, the number who go on to consent and those who either choose not to take part (including reasons given) and those who are ruled out on eligibility grounds
- Take-up, attendance and engagement with the PAT-A© intervention and CCSP
- Completion of follow-up data at 3 months and 12 months post intervention
- Qualitative data regarding the acceptability of PAT-A© from the participants’ perspective

**Fig. 3:**
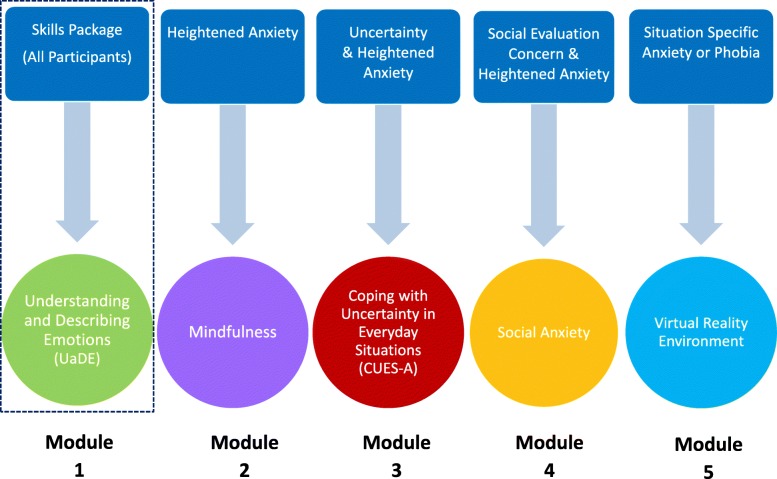
Personalised Anxiety Treatment–Autism (PAT-A©) treatment modules*.* Note: the number of sessions devoted to any PAT-A package depends upon the participant’s formulation, treatment plan, and the ability to engage in the therapeutic process

We will also undertake some preliminary analysis of treatment effects, and follow a pre-existing statistical analysis plan. The difference in mean change as assessed by the main outcome measure from our previous trials (‘target-situation’ vignettes) and anxiety screening tools will be examined between the groups from baseline to 3 months post intervention with accompanying 95% confidence intervals. Results will be interpreted cautiously because of the size of the study, the possible imbalance in pre-randomisation baseline covariates and the range of interventions offered to participants within the CCSP arm of the trial (Fig. 3).

### Dissemination plan

Dissemination of the trial findings will be undertaken in several ways. We will work closely with autistic people, who will review the findings alongside members of the research team and assist with ensuring that the results are interpreted and reported in a meaningful way. A short webinar about the findings will be created and hosted on our website(s) with links to this shared within dissemination events and with key local and national stakeholders. A series of reports will be written in accessible language and will include ‘EasyRead’ versions to maximise accessibility. We will prepare newsletters/reports for autistic people and their families, professionals, commissioners and other key agencies. Results will be presented at academic meetings and research papers submitted to open-access, peer-reviewed scientific journals.

## Discussion

### Significance

The PAT-A© intervention is a novel approach to treating anxiety conditions in autistic adults. It is a highly individualised, modular and formulation-driven psychological therapy that aims to improve both the understanding and management of emotions and by targeting one or more situations salient to the participant, to improve functioning and reduce anxiety. If this approach to anxiety treatment in autistic adults is found to be acceptable (to autistic people and professionals) and effective it could be used widely in healthcare settings and thus improve everyday functioning and quality of life for affected adults and reduce the burden of care for individuals, families and the wider community. Study outcomes will be used to inform an application for a fully powered multi-site intervention trial of adults and young people. 

### Strengths and limitations

The CCSP (control) arm of this trial consists of usual care from local services with two additional sessions focussing upon emotional recognition skills. The decision to offer an enhanced level of care reflects feedback from the autistic community about the acceptability of trial methods. This enhanced package may support retention of participants who are randomised to the control arm and will provide a stringent test in a fully powered trial.

Our primary method of recruitment potentially excludes adults who struggle to, or do not, engage with primary care or mental health services [58]; however, consideration of methods to include participants from other settings is beyond the scope of this feasibility trial, which is focussed on recruitment from mental health services. Should the PAT-A© intervention demonstrate efficacy in a fully powered trial, then a next step would be to consider the development of a modified treatment package for autistic people who may require additional support to access services.

### Trial status

Protocol Version 2.0 (1 March 2019). The first participant was recruited in November 2018. At the time of submission, the PAT-A© trial is currently recruiting. Recruitment is expected to continue until November 2019. The PAT-A© trial is monitored by the sponsoring NHS trust. The trial is registered with ISRCTN and the NIHR Portfolio.

### Trial sponsorship

The PAT-A trial is sponsored by Cumbria, Northumberland, Tyne and Wear NHS Foundation Trust. Address: St Nicholas Hospital, Jubilee Road, Gosforth, Newcastle upon Tyne, Tyne and Wear, NE3 3XT. Tel: 0191 2081356. Web: www.cntw.nhs.uk

## Data Availability

Anonymised datasets used and/or analysed may be available from the corresponding author upon reasonable request. If the research data shows acceptability and efficacy, the PAT-A© treatment manuals will be available from the authors in the future. The final trial dataset (anonymised) will otherwise only be accessible by the Trial Management Team led by the co-lead investigators (JP and JR).

## Abbreviations

ADIS-5: Anxiety and Related Disorders Interview Schedule for *DSM-5*: Adult Version
ASA-A: Anxiety Scale for Autism - Adults
ASC: Autism Spectrum Condition (please note: we use the this term throughout the paper to refer to any Autism Spectrum Condition including Asperger’s syndrome)
CBT: Cognitive behavioural therapy
CUES: Coping with Uncertainty in Everyday Situations
HADS: Hospital Anxiety and Depression Scale;
NHS: National Health Service
NICE: National Institute for Health and Care Excellence
PAT-A©: Personalised Anxiety Treatment in Autism–Adult
SRS-2: Social Responsiveness Scale
TAS-20: Toronto Alexithymia Scale-20
UK: United Kingdom
VREs: Virtual Reality Environments (VREs)
W-ADL: Waisman Activities of Daily Living Scale
WHOQOL-BREF: World Health Organisation Quality of Life-BREF

## References

[1] Association AP (2013). Diagnostic and Statistical Manual of Mental Disorders.

[2] Matson JL, Williams LW (2013). Differential diagnosis and comorbidity: distinguishing autism from other mental health issues. Neuropsychiatry.

[3] Croen LA, Zerbo O, Qian Y, Massolo ML, Rich S, Sidney S (2015). The health status of adults on the autism spectrum. Autism.

[4] Lugnegård T, Hallerbäck MU, Gillberg C (2011). Psychiatric comorbidity in young adults with a clinical diagnosis of Asperger syndrome. Res Dev Disabil.

[5] Buck TR, Viskochil J, Farley M, Coon H, McMahon WM, Morgan J (2014). Psychiatric comorbidity and medication use in adults with autism spectrum disorder. J Autism Dev Disord.

[6] Joshi G, Wozniak J, Petty C, Martelon MK, Fried R, Bolfek A (2013). Psychiatric comorbidity and functioning in a clinically referred population of adults with autism spectrum disorders: a comparative study. J Autism Dev Disord.

[7] Kerns CM, Kendall PC, Berry L, Souders MC, Franklin ME, Schultz RT (2014). Traditional and atypical presentations of anxiety in youth with autism spectrum disorder. J Autism Dev Disord.

[8] World Health Organization (2001). International Classification of Functioning, Disability and Health (ICF).

[9] de Schipper E, Mahdi S, de Vries P, Granlund M, Holtmann M, Karande S (2016). Functioning and disability in autism spectrum disorder: a worldwide survey of experts. Autism Res.

[10] Mason D, Mackintosh J, McConachie H, Rodgers J, Finch T, Parr JR (2019). Quality of life for older autistic people: the impact of mental health difficulties. Res Autism Spectr Disord.

[11] Griffiths S, Allison C, Kenny R, Holt R, Smith P, Baron-Cohen S (2019). The Vulnerability Experiences Quotient (VEQ): a study of vulnerability, mental health and life satisfaction in autistic adults. Autism Res.

[12] Lipinski S, Blanke ES, Suenkel U, Dziobek I. Outpatient psychotherapy for adults with high-functioning autism spectrum condition: utilization, treatment satisfaction, and preferred modifications. J Autism Dev Disord. 2018;49(3):1154–1168. 10.1007/s10803-018-3797-1 30415320

[13] Hallett SC, Crompton CJ (2018). Too complicated to treat? Autistic people seeking mental health support in Scotland.

[14] Camm-Crosbie L, Bradley L, Shaw R, Baron-Cohen S, Cassidy S. ‘People like me don’t get support’: autistic adults’ experiences of support and treatment for mental health difficulties, self-injury and suicidality. Autism. 0(0):1362361318816053. 10.1177/1362361318816053 PMC6625034 30497279

[15] National Institute for Health and Care Excellence (2011). Common mental health problems: identification and pathways to care. Clinical guideline [CG123].

[16] National Institute for Health and Care Excellence (2011). Generalised anxiety disorder and panic disorder in adults: management: Clinical guideline [CG113].

[17] Spain D, Sin J, Chalder T, Murphy D, Happé F (2015). Cognitive behaviour therapy for adults with autism spectrum disorders and psychiatric co-morbidity: a review. Res Autism Spectr Disord.

[18] Brunsdon VEA, Happé F (2013). Exploring the ‘fractionation’ of autism at the cognitive level. Autism.

[19] Harris PL, Leevers HJ (2000). Pretending, imagery and self-awareness in autism. Understanding other minds: perspectives from developmental cognitive neuroscience.

[20] Gaus VL (2011). Adult Asperger syndrome and the utility of cognitive-behavioral therapy. J Contemp Psychother.

[21] Mason D, Ingham B, Urbanowicz A, Michael C, Birtles H, Woodbury-Smith M, et al. A systematic review of what barriers and facilitators prevent and enable physical healthcare services access for autistic adults. J Autism Dev Disord. 2019;49(8):3387–3400. 10.1007/s10803-019-04049-2 PMC6647496 31124030

[22] South M, Rodgers J (2017). Sensory, emotional and cognitive contributions to anxiety in autism spectrum disorders. Front Hum Neurosci.

[23] Trembath D, Germano C, Johanson G, Dissanayake C (2012). The experience of anxiety in young adults with autism spectrum disorders. Focus Autism Other Dev Disabil.

[24] Simon Wallace JPAH (2013). One in a hundred: putting families at the heart of autism research.

[25] James Lind Alliance (2016). Autism Top Ten.

[26] Social Care LGaCPD, Department of Health. Think Autism: Fulfilling and Rewarding Lives, the strategy for adults with autism in England: an update. London: Department of Health; 2014.

[27] National Institute for Health and Care Excellence (2012). Autism spectrum disorder in adults: diagnosis and management. Clinical guideline [CG142].

[28] White SW, Simmons GL, Gotham KO, Conner CM, Smith IC, Beck KB (2018). Psychosocial treatments targeting anxiety and depression in adolescents and adults on the autism spectrum: review of the latest research and recommended future directions. Curr Psychiatry Rep.

[29] Cardaciotto L, Herbert JD (2004). Cognitive behavior therapy for social anxiety disorder in the context of Asperger's syndrome: a single-subject report. Cogn Behav Pract.

[30] Turner MA, Hammond N (2016). Cognitive behavioural therapy in the treatment of social skills deficits and social phobia in a man with an autism spectrum disorder: a single-case study. Cogn Behav Ther.

[31] Weiss JA, Lunsky Y (2010). Group cognitive behaviour therapy for adults with Asperger syndrome and anxiety or mood disorder: a case series. Clin Psychol Psychother.

[32] Langdon PE, Murphy GH, Shepstone L, Wilson ECF, Fowler D, Heavens D (2018). The People with Asperger Syndrome and Anxiety disorders (PAsSA) trial: a pilot multicentre, single-blind randomised trial of group cognitive-behavioural therapy. BJPsych Open.

[33] Maskey M, Rodgers J, Ingham B, Freeston M, Evans G, Labus M, Parr JR (2019). Using virtual reality environments to augment cognitive behavioural therapy for fears and phobias in autistic adults. Autism Adulthood.

[34] Kiep M, Spek AA, Hoeben L (2015). Mindfulness-based therapy in adults with an autism spectrum disorder: do treatment effects last?. Mindfulness.

[35] Sizoo BB, Kuiper E (2017). Cognitive behavioural therapy and mindfulness based stress reduction may be equally effective in reducing anxiety and depression in adults with autism spectrum disorders. Res Dev Disabil.

[36] Boulter C, Freeston M, South M, Rodgers J (2014). Intolerance of uncertainty as a framework for understanding anxiety in children and adolescents with autism spectrum disorders. J Autism Dev Disord.

[37] Chamberlain PD, Rodgers J, Crowley MJ, White SE, Freeston MH, South M (2013). A potentiated startle study of uncertainty and contextual anxiety in adolescents diagnosed with autism spectrum disorder. Mol Autism.

[38] Wigham S, Rodgers J, South M, McConachie H, Freeston M (2015). The interplay between sensory processing abnormalities, intolerance of uncertainty, anxiety and restricted and repetitive behaviours in autism spectrum disorder. J Autism Dev Disord.

[39] Keefer A, Kreiser NL, Singh V, Blakeley-Smith A, Duncan A, Johnson C (2017). Intolerance of uncertainty predicts anxiety outcomes following CBT in youth with ASD. J Autism Dev Disord.

[40] Rodgers J, Herrema R, Honey E, Freeston M (2018). Towards a treatment for intolerance of uncertainty for autistic adults: a single case experimental design study. J Autism Dev Disord.

[41] Lancaster GA, Dodd S, Williamson PR (2004). Design and analysis of pilot studies: recommendations for good practice. J Eval Clin Pract.

[42] Gu J, Strauss C, Bond R, Cavanagh K (2015). How do mindfulness-based cognitive therapy and mindfulness-based stress reduction improve mental health and wellbeing? A systematic review and meta-analysis of mediation studies. Clin Psychol Rev.

[43] Conner CM, White SW (2018). Brief report: feasibility and preliminary efficacy of individual mindfulness therapy for adults with autism spectrum disorder. J Autism Dev Disord.

[44] Rodgers J, Hodgson A, Shields K, Wright C, Honey E, Freeston M (2017). Towards a treatment for intolerance of uncertainty in young people with autism spectrum disorder: development of the Coping with Uncertainty in Everyday Situations (CUES©) programme. J Autism Dev Disord.

[45] National Institute for Health and Care Excellence (2013). Social anxiety disorder: recognition, assessment and treatment. Clinical Guideline [CG159].

[46] Maskey M, Lowry J, Rodgers J, McConachie H, Parr JR (2014). Reducing specific phobia/fear in young people with Autism Spectrum Disorders (ASDs) through a virtual reality environment intervention. PLoS One.

[47] Maskey M, McConachie H, Rodgers J, Grahame V, Maxwell J, Tavernor L (2019). An intervention for fears and phobias in young people with autism spectrum disorders using flat screen computer-delivered virtual reality and cognitive behaviour therapy. Res Autism Spectr Disord.

[48] Brown TA, Barlow DH (2014). Anxiety and Related Disorders Interview Schedule for DSM-5 (ADIS-5L)-lifetime version: client interview schedule 5-copy set (treatments that work).

[49] Arnold LE, Vitiello B, McDougle C, Scahill L, Shah B, Gonzalez NM (2003). Parent-defined target symptoms respond to risperidone in RUPP autism study: customer approach to clinical trials. J Am Acad Child Adolesc Psychiatry.

[50] Maskey M, Rodgers J, Grahame V, Glod M, Honey E, Kinnear J (2019). A randomised controlled feasibility trial of immersive virtual reality treatment with cognitive behaviour therapy for specific phobias in young people with autism spectrum disorder. J Autism Dev Disord.

[51] Maskey M, Rodgers J, Ingham B, Freeston M, Evans G, Labus M (2019). Using virtual reality environments to augment cognitive behavioral therapy for fears and phobias in autistic adults. Autism Adulthood.

[52] McConachie H, McLaughlin E, Grahame V, Taylor H, Honey E, Tavernor L (2014). Group therapy for anxiety in children with autism spectrum disorder. Autism.

[53] Gruber JNCCP (2012). Social Responsiveness Scale-Second Edition (SRS-2).

[54] Mandell DS, Lawer LJ, Branch K, Brodkin ES, Healey K, Witalec R (2012). Prevalence and correlates of autism in a state psychiatric hospital. Autism.

[55] Rodgers J, Farquhar K, Mason D, Brice S, Wigham S, Ingham B, et al. Development and initial evaluation of the Anxiety Scale for Autism–Adult (ASA-A). 10.1089/aut.2019.0044. 10.1089/aut.2019.0044 PMC8992845 36600985

[56] Rodgers J, Wigham S, McConachie H, Freeston M, Honey E, Parr JR (2016). Development of the Anxiety SCale for children with Autism Spectrum Disorder (ASC-ASD). Autism Res.

[57] Uljarević M, Richdale AL, McConachie H, Hedley D, Cai RY, Merrick H (2018). The Hospital Anxiety and Depression Scale: factor structure and psychometric properties in older adolescents and young adults with autism spectrum disorder. Autism Res.

[58] The WHOQOL Group (1998). Development of the World Health Organization WHOQOL-BREF Quality of Life Assessment. Psychol Med.

[59] Ayres M, Parr JR, Rodgers J, Mason D, Avery L, Flynn D (2018). A systematic review of quality of life of adults on the autism spectrum. Autism.

[60] Power MJ, Green AM, The W-DG (2010). Development of the WHOQOL disabilities module. Qual Life Res.

[61] McConachie H, Mason D, Parr JR, Garland D, Wilson C, Rodgers J (2018). Enhancing the validity of a quality of life measure for autistic people. J Autism Dev Disord.

[62] Herdman M, Gudex C, Lloyd A, Janssen MF, Kind P, Parkin D (2011). Development and preliminary testing of the new five-level version of EQ-5D (EQ-5D-5L). Qual Life Res.

[63] Janssen MF, Pickard AS, Golicki D, Gudex C, Niewada M, Scalone L (2013). Measurement properties of the EQ-5D-5L compared to the EQ-5D-3L across eight patient groups: a multi-country study. Qual Life Res.

[64] Bagby RM, Parker JDA, Taylor GJ (1994). The twenty-item Toronto Alexithymia Scale—I. Item selection and cross-validation of the factor structure. J Psychosom Res.

[65] Berthoz S, Hill EL (2005). The validity of using self-reports to assess emotion regulation abilities in adults with autism spectrum disorder. Eur Psychiatry.

[66] Bell ML, Fairclough DL, Fiero MH, Butow PN (2016). Handling missing items in the Hospital Anxiety and Depression Scale (HADS): a simulation study. BMC Res Notes.

[67] Linden M (2013). How to define, find and classify side effects in psychotherapy: from unwanted events to adverse treatment reactions. Clin Psychol Psychother.

